# Explore how immobilization strategies affected immunosensor performance by comparing four methods for antibody immobilization on electrode surfaces

**DOI:** 10.1038/s41598-022-26768-w

**Published:** 2022-12-23

**Authors:** Jiaoling Huang, Zhixun Xie, Liji Xie, Sisi Luo, Tingting Zeng, Yanfang Zhang, Minxiu Zhang, Sheng Wang, Meng Li, You Wei, Qing Fan, Zhiqin Xie, Xianwen Deng, Dan Li

**Affiliations:** Guangxi Key Laboratory of Veterinary Biotechnology, Key Laboratory of China (Guangxi)-ASEAN Cross-Border Animal Disease Prevention and Control, Ministry of Agriculture and Rural Affairs of China, Guangxi Veterinary Research Institute, Nanning, Guangxi China

**Keywords:** Chemical biology, Immunology

## Abstract

Among the common methods used for antibody immobilization on electrode surfaces, which is the best available option for immunosensor fabrication? To answer this question, we first used graphene-chitosan-Au/Pt nanoparticle (G-Chi-Au/PtNP) nanocomposites to modify a gold electrode (GE). Second, avian reovirus monoclonal antibody (ARV/MAb) was immobilized on the GE surface by using four common methods, which included glutaraldehyde (Glu), 1-ethyl-3-(3-dimethylaminopropyl)-carbodiimide/*N*-hydroxysuccinimide (EDC/NHS), direct incubation or cysteamine hydrochloride (CH). Third, the electrodes were incubated with bovine serum albumin, four different avian reovirus (ARV) immunosensors were obtained. Last, the four ARV immunosensors were used to detect ARV. The results showed that the ARV immunosensors immobilized via Glu, EDC/NHS, direct incubation or CH showed detection limits of 10^0.63^ EID_50_ mL^−1^, 10^0.48^ EID_50_ mL^−1^, 10^0.37^ EID_50_ mL^−1^ and 10^0.46^ EID_50_ mL^−1^ ARV (S/N = 3) and quantification limits of 10^1.15^ EID_50_ mL^−1^, and 10^1.00^ EID_50_ mL^−1^, 10^0.89^ EID_50_ mL^−1^ and 10^0.98^ EID_50_ mL^−1^ ARV (S/N = 10), respectively, while the linear range of the immunosensor immobilized via CH (0–10^5.82^ EID_50_ mL^−1^ ARV) was 10 times broader than that of the immunosensor immobilized via direct incubation (0–10^4.82^ EID_50_ mL^−1^ ARV) and 100 times broader than those of the immunosensors immobilized via Glu (0–10^3.82^ EID_50_ mL^−1^ ARV) or EDC/NHS (0–10^3.82^ EID_50_ mL^−1^ ARV). And the four immunosensors showed excellent selectivity, reproducibility and stability.

## Introduction

Electrochemical immunosensors are a novel detection method that are combined with electrochemical analysis and immunoassays and are widely used in the diagnosis of various pathogens due to their advantages of simplicity, rapidity, and high sensitivity in point-of-care testing^1,2^. The sensitivity and linear range of electrochemical immunosensors are affected by the electrode surface modification and antibody immobilization strategy^3,4^. Electrochemical immunosensors can be categorized as “label-free” and “sandwich” types^5,6^. “Sandwich” immunosensors waste time and energy compared to the “label-free” type, in which the electrochemical signal molecule is immobilized on the electrode or dissolved in an electrolyte in only one step to detect the target^7,8^. Therefore, it is more desirable to construct “label-free” electrochemical immunosensors.

Substrate materials with large specific surface areas and high conductivities are important in improving the capabilities of electrochemical immunosensors. The large specific surface area enhances antibody surface loading^9^, and the high conductivity enhances electron transfer from the target molecules to the surface of electrode^10^. Different nanoparticles with large specific surface areas and high electronic transmission capabilities were used to modify electrode surfaces and to increase the sensitivity and linear range of “label-free” electrochemical immunosensors, and this approach has been reported many times^11,12^. Graphene (G) has shown potential for application in electrochemical immunosensors because of its low manufacturing cost, superior conductivity and large specific surface area^13^. Among metal nanomaterials, gold nanoparticles (AuNPs) and platinum nanoparticles (PtNPs) are most widely used in electrochemical immunosensors because of their excellent performance and excellent conductivity^14,15^. Additionally, G has been functionalized and adsorbed on chitosan (Chi) through π–π stacking to form graphene-chitosan (G-Chi) hybrid materials^16^. In the G-Chi hybrid materials, Chi chelates Au^3+^ and Pt^2+^ metal ions and acts as a reducing agent to convert the Au^3+^ and Pt^2+^ ions into AuNPs and PtNPs^17,18^; further, G-Chi hybrid materials can be loaded with substantial amounts of AuNPs and PtNPs because of the large specific surface area of G. Hence, we designed a “label-free” electrochemical immunosensor based on G-Chi-Au/PtNP nanocomposites in this work.

In addition, effective immobilization of antibodies is an essential step in constructing electrochemical immunosensors and constitute another important factor in improving the performance of the electrochemical immunosensors^19^. The sensitivities and linear ranges of electrochemical immunosensors are limited by the antibody immobilization strategy chosen^20^. Various antibody immobilization strategies, including glutaraldehyde (Glu) cross-linking, 1-ethyl-3-(3-dimethylaminopropyl)-carbodiimide/*N*-hydroxysuccinimide (EDC/NHS) chemistry, direct incubation and cysteamine hydrochloride (CH), have been exploited by different research groups^21–26^, but it is not known which approach is best. To answer this question, four immobilization strategies were compared in the present study: (1) Glu immobilization, (2) EDC/NHS immobilization, (3) direct immobilization and (4) CH immobilization. The results showed that the linear range obtained with the CH immobilization strategy was 10 times broader than that realized with the direct immobilization strategy and 100 times broader than those seen with the Glu immobilization strategy and EDC/NHS immobilization strategy, and their detection limits were similar.

## Results and discussion

### Nanoparticle synthesis and characterization

**Figure 1:**
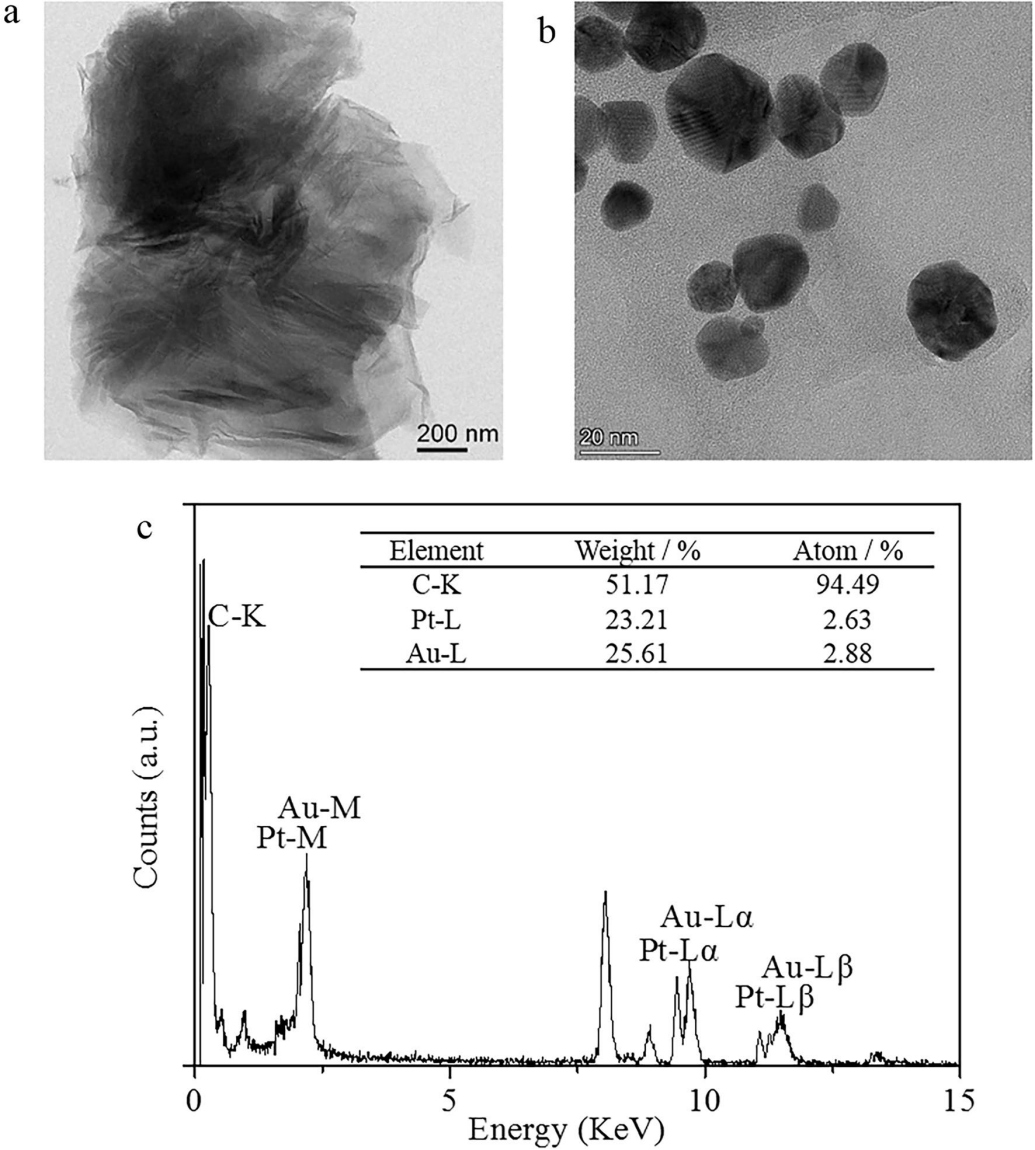
Characterization of the investigated nanocomposites. TEM images of G-Chi (**a**) and G-Chi-Au/PtNP (**b**). EDS elemental analysis of G-Chi-Au/PtNP (**c**).

A transmission electron microscopy (TEM) micrograph of G-Chi, which has a thin, wrinkled, rippled and flake-like structure, is shown in Fig. 1a. Figure 1b shows the TEM micrograph of G-Chi-Au/PtNP, which indicates that Au/Pt was loaded on the surface of G-Chi. In addition, energy dispersive spectroscopy (EDS) elemental analysis of G-Chi-Au/PtNP was employed to determine the presence of Au and Pt, which confirmed that Au/PtNP had been loaded on the surface of G-Chi (Fig. 1c). The mechanism for formation of G-Chi-Au/PtNP involved Au^3+^ and Pt^2+^ adsorption from aqueous solution due to chelation by G-Chi and then reduction to Au/Pt nanoparticles by Chi. Chi was used as both a stabilizing agent and reducing agent.

### Electrochemical characterization

**Figure 2:**
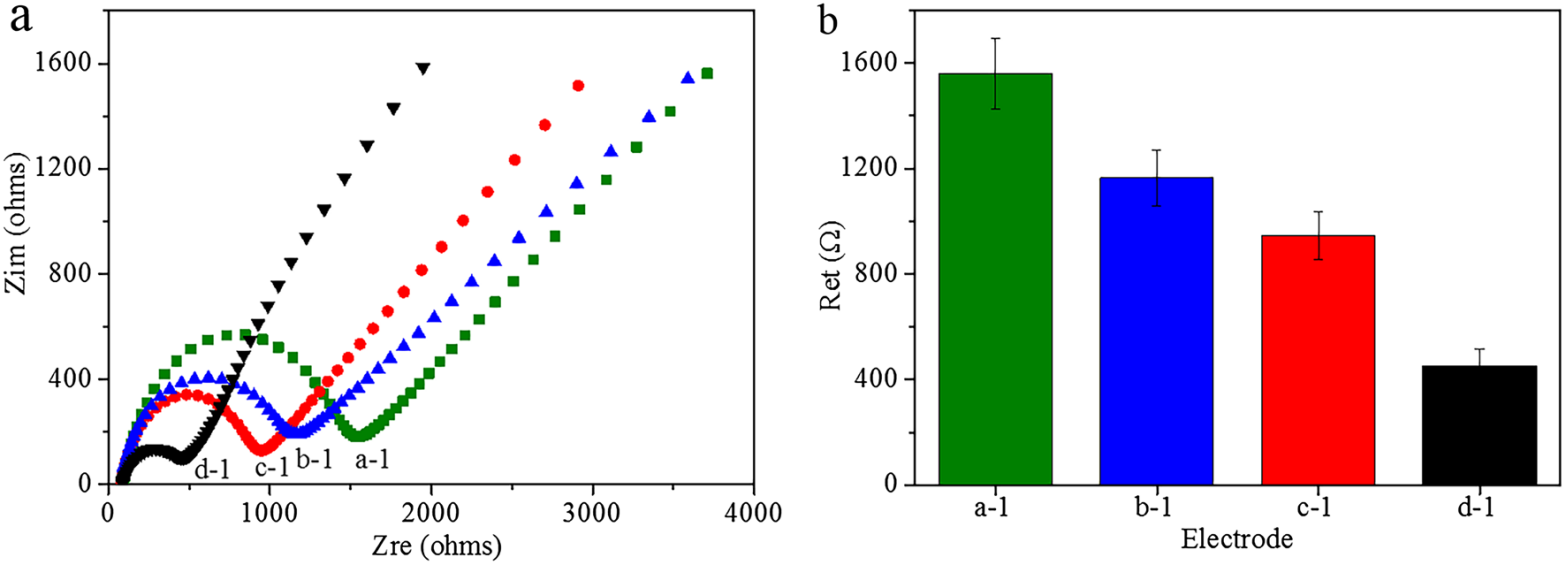
(**a**) Nyquist plots and (**b**) semicircle diameters from EIS characterization of electrodes modified with different materials in 0.01 M PBS (pH = 7.0) containing 5 mM [Fe(CN)_6_]^3−/4−^ and 0.1 M KCl: (a-1) GE, (b-1) GE-G-Chi-PtNP, (c-1) GE-G-Chi-AuNP, (d-1) GE-G-Chi-Au/PtNP. Error bar = RSD ( n = 5).

Electrochemical impedance spectroscopy (EIS) is an effective technique for probing the features of surface modified electrodes, and it sensitively analyzes the interactions of analytes with modified electrodes and produces measurable electric signals. More important, EIS is more sensitive than either amperometric or voltammetric methods^27,28^. In typical EIS Nyquist plots, a semicircle appears in the high-frequency region, while a line appears in the low-frequency region, and the diameter of the semicircle corresponds to the electron transfer resistance (R_et_). In brief, the resistance on the surface of the electrode can be estimated by determining the semicircle diameter. Here, EIS was employed to characterize which material is better for electrode modification, and the results are shown in Fig. 2. The diameter of the semicircle in the Nyquist plot of GE corresponds to an impedance of 1561 Ω, which was decreased to 1167 Ω, 950 Ω and 439 Ω upon modification of the GE with G-Chi-PtNP, G-Chi-AuNP, and G-Chi-Au/PtNP, respectively, due to the high conductivities of G, AuNP and PtNP. These results showed that G-Chi-Au/PtNP exhibited the fastest electron transfer, and it was selected as the material for GE modification.

**Figure 3:**
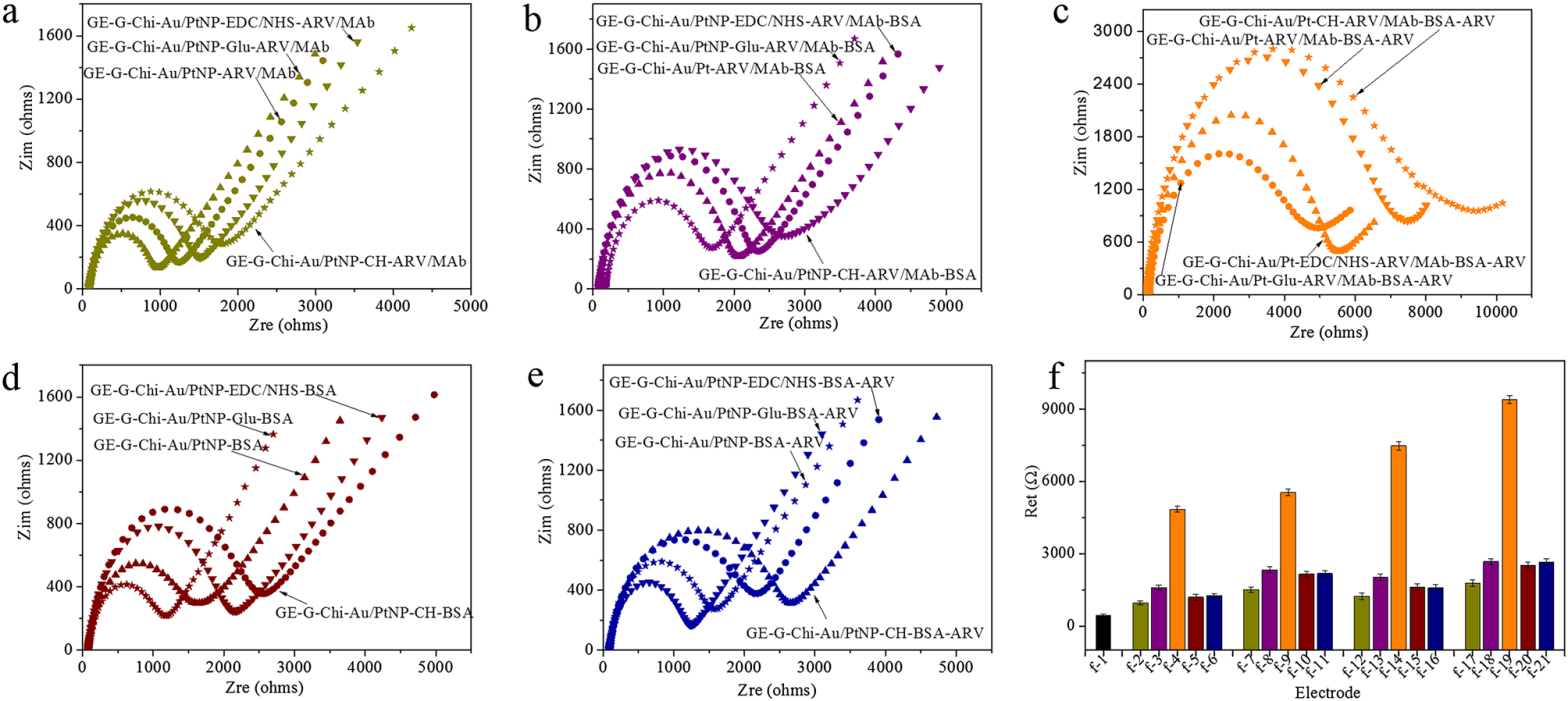
Nyquist plots for EIS characterization of electrodes after different modification steps in electrolyte (pH = 7.0) containing 5 mM [Fe(CN)_6_]^3−/4−^: (**a**) after ARV/MAb immobilization on GE-G-Chi-Au/PtNP, (**b**) after blocking with BSA, (**c**) after incubation with 10^6.82^ EID_50_ mL^−1^ ARV, (**d**) after blocking with BSA without ARV/MAb immobilization, (**e**) after incubation with 10^6.82^ EID_50_ mL^−1^ ARV without ARV/MAb immobilization; (**f**) R_et_ from EIS characterization of electrodes at different modification steps in electrolyte (pH = 7.0) containing 5 mM [Fe(CN)_6_]^3−/4−^: (f-1) GE-G-Chi-Au/PtNP, (f-2) GE-G-Chi-Au/PtNP-Glu-ARV/MAb, (f-3) GE-G-Chi-Au/PtNP-Glu-ARV/MAb-BAS, (f-4) GE-G-Chi-Au/PtNP-Glu-ARV/MAb-BAS-ARV, (f-5) GE-G-Chi-Au/PtNP-Glu-BAS, (f-6) GE-G-Chi-Au/PtNP-Glu-BAS-ARV, (f-7) GE-G-Chi-Au/PtNP-EDC/NHS-ARV/MAb, (f-8) GE-G-Chi-Au/PtNP-EDC/NHS-ARV/MAb-BSA, (f-9) GE-G-Chi-Au/PtNP-EDC/NHS-ARV/MAb-BSA-ARV, (f-10) GE-G-Chi-Au/PtNP-EDC/NHS-BSA, (f-11) GE-G-Chi-Au/PtNP-EDC/NHS-BSA-ARV, (f-12) GE-G-Chi-Au/PtNP-ARV/MAb, (f-13) GE-G-Chi-Au/PtNP-ARV/MAb-BSA, (f-14) GE-G-Chi-Au/PtNP-ARV/MAb-BSA-ARV, (f-15) GE-G-Chi-Au/PtNP-BSA, (f-16) GE-G-Chi-Au/PtNP-BSA-ARV, (f-17) GE-G-Chi-Au/PtNP-CH-ARV/MAb, (f-18) GE-G-Chi-Au/PtNP-CH-ARV/MAb-BSA, (f-19) GE-G-Chi-Au/PtNP-CH-ARV/MAb-BSA-ARV, (f-20) GE-G-Chi-Au/PtNP-CH-BSA, (f-21) GE-G-Chi-Au/PtNP-CH-BSA-ARV. Error bar = RSD (n = 5).

In addition, EIS was employed to survey the layer-by-layer modification of GE. Figure 3a and f shows that after the ARV/MAb was immobilized on GE-G-Chi-Au/PtNP by four different methods, the R_et_s of the GE-G-Chi-Au/PtNP-Glu-ARV/MAb, GE-G-Chi-Au/PtNP-ARV/MAb, GE-G-Chi-Au/PtNP-EDC/NHS-ARV/MAb and GE-G-Chi-Au/PtNP-CH-ARV/MAb increased to 965 Ω, 1232 Ω, 1519 Ω and 1778 Ω, respectively, because ARV/MAb is protein with poor electrical conductivity and impedes electron transfer to the surface of the electrode. After GE-G-Chi-Au/PtNP-Glu-ARV/MAb, GE-G-Chi-Au/PtNP-ARV/MAb, GE-G-Chi-Au/PtNP-EDC/NHS-ARV/MAb and GE-G-Chi-Au/PtNP-CH-ARV/MAb were blocked with BSA, their R_et_ values were further increased to 1598 Ω, 2078 Ω, 2331 Ω and 2670 Ω, respectively (Fig. 3b,f), because BSA is a protein. More important, these results show that the ability of the four different methods for ARV/MAb immobilization decreased in the order CH > EDC/NHS > direct incubation > Glu. After GE-G-Chi-Au/PtNP-Glu-ARV/MAb-BSA, GE-G-Chi-Au/PtNP-ARV/MAb-BSA, GE-G-Chi-Au/PtNP-EDC/NHS-ARV/MAb-BSA and GE-G-Chi-Au/PtNP-CH-ARV/MAb-BSA were incubated with 10^6.82^ EID_50_ mL^−1^ ARV, the ARV was adsorbed on the electrodes via a specific response with ARV/MAb, and the corresponding R_et_s were further increased to 4841 Ω, 7468 Ω, 5547 Ω, and 9417 Ω (Fig. 3c,f) because electron transfer to the surface of the electrode was impeded by the ARV protein, and the results demonstrated that the ability of the four different immunosensors to combine ARV decreased in the order CH > direct incubation > EDC/NHS > Glu. Most importantly, the different relative orders for ARV/MAb immobilization and the ARV immobilization demonstrated that the number of ARVs adsorbed on the immunosensors was not only related to the number of ARVs/MAbs but was also affected by the method used to immobilize the ARVs/MAbs.

In addition, BSA was immobilized on GE-G-Chi-Au/PtNP by four different methods to evaluate the ability of the four methods to immobilize protein again, the results are shown in Fig. 3d and f. The R_et_s of the GE-G-Chi-Au/PtNP-Glu-BSA, GE-G-Chi-Au/PtNP-BSA, GE-G-Chi-Au/PtNP-EDC/NHS-BSA and GE-G-Chi-Au/PtNP-CH-BSA were increased to 1196 Ω, 1613 Ω, 2159 Ω and 2537 Ω because the BSA protein adsorbed on the surface of GE-G-Chi-Au/PtNP impeded electron transfer to the surface of the electrode. These results showed that the abilities of the four different methods to immobilize BSA decreased in the order CH > EDC/NHS > direct incubation > Glu, which corresponds to the results for ARV/MAb immobilization on GE-G-Chi-Au/PtNP by the four different methods. To evaluate the specificities of the immunosensors, GE-G-Chi-Au/PtNP-Glu-BSA, GE-G-Chi-Au/PtNP-BSA, GE-G-Chi-Au/PtNP-EDC/NHS-BSA and GE-G-Chi-Au/PtNP-CH-BSA (which were not yet immobilized with ARV/MAb) were incubated with 10^6.82^ EID_50_ mL^−1^ ARV, the results were showed in Fig. 3e and f. The results showed that the values of R_et_s of GE-G-Chi-Au/PtNP-Glu-BSA, GE-G-Chi-Au/PtNP-BSA, GE-G-Chi-Au/PtNP-EDC/NHS-BSA and GE-G-Chi-Au/PtNP-CH-BSA were hardly change after incubation with ARV, which suggested that the GE-G-Chi-Au/PtNP-Glu-ARV/MAb-BSA, GE-G-Chi-Au/PtNP-ARV/MAb-BSA, GE-G-Chi-Au/PtNP-EDC/NHS-ARV/MAb-BSA and GE-G-Chi-Au/PtNP-CH-ARV/MAb-BSA had high specificities.

**Figure 4:**
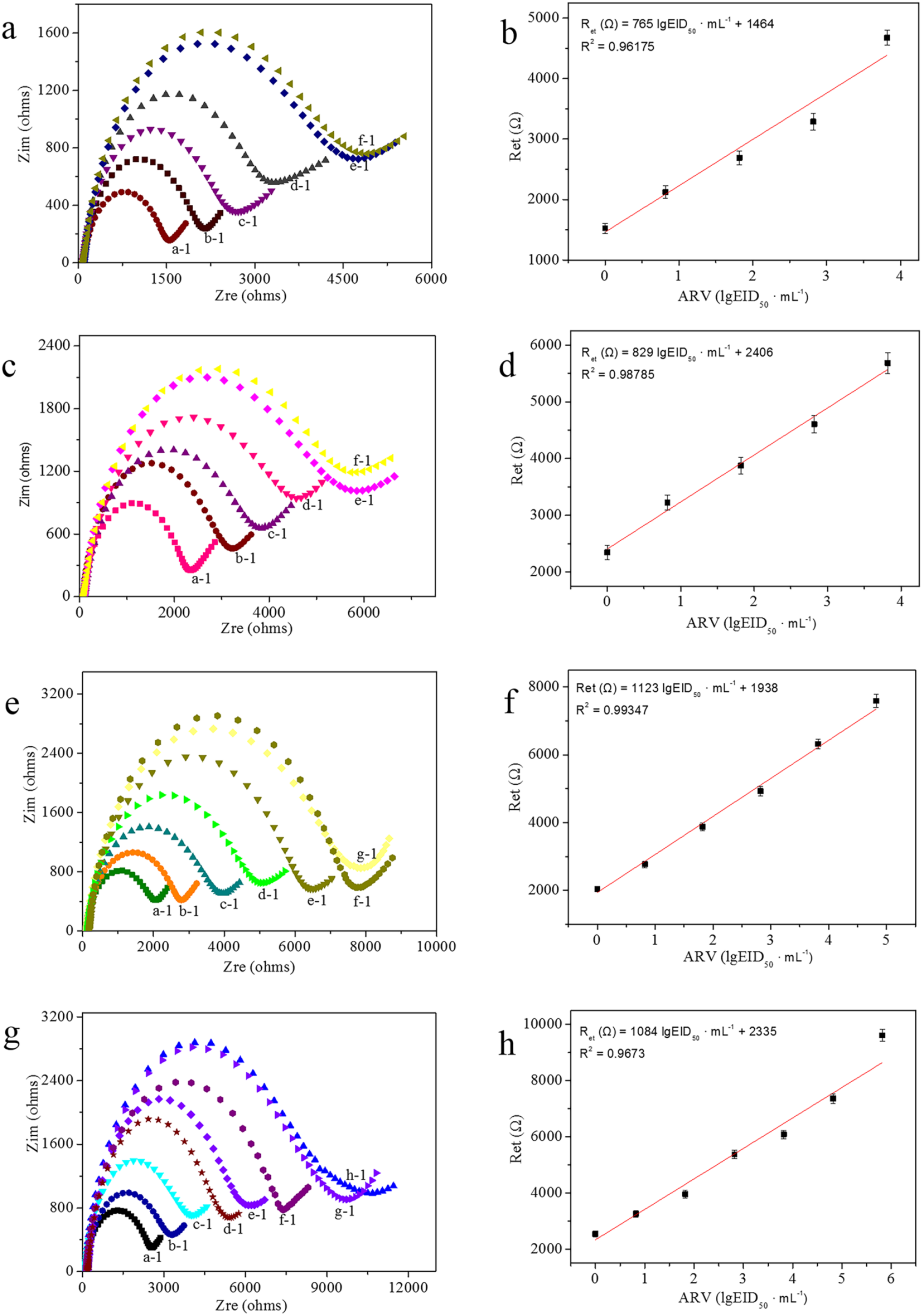
(**a**) Nyquist plots for different concentrations of ARV on GE-G-Chi-Au/PtNP-Glu-ARV/MAb-BSA; (**b**) Calibration curve of GE-G-Chi-Au/PtNP-Glu-ARV/MAb-BSA with different concentrations of ARV. Error bar = RSD (n = 5). (**c**) Nyquist plots for different concentrations of ARV on GE-G-Chi-Au/PtNP-EDC/NHS-ARV/MAb-BSA; (**d**) Calibration curve of GE-G-Chi-Au/PtNP-EDC/NHS-ARV/MAb-BSA with different concentrations of ARV. Error bar = RSD (n = 5). (**e**) Nyquist plots for different concentrations of ARV on GE-G-Chi-Au/PtNP-ARV/MAb-BSA; (**f**) Calibration curve for GE-G-Chi-Au/PtNP-ARV/MAb-BSA with different concentrations of ARV. Error bar = RSD (n = 5). (**g**) Nyquist plots for different concentrations of ARV on GE-G-Chi-Au/PtNP-CH-ARV/MAb-BSA; (**h**) Calibration curve for GE-G-Chi-Au/PtNP-CH-ARV/MAb-BSA with different concentrations of ARV. Error bar = RSD (n = 5). (a-1) 0, (b-1) 10^0.82^ EID_50_ mL^−1^ ARV, (c-1) 10^1.82^ EID_50_ mL^−1^ ARV, (d-1) 10^2.82^ EID_50_ mL^−1^ ARV, (e-1) 10^3.82^ EID_50_ mL^−1^ ARV, (f-1) 10^4.82^ EID_50_ mL^−1^ ARV, (g-1) 10^5.82^ EID_50_ mL^−1^ ARV, (f-1) 10^6.82^ EID_50_ mL^−1^ ARV.

Sensitivity, saturation and extended dynamic range are important factors used to evaluate the performance of an immunosensor. To obtain this information for the immunosensors, the GE-G-Chi-Au/PtNP-Glu-ARV/MAb-BSA, GE-G-Chi-Au/PtNP-EDC/NHS-ARV/MAb-BSA, GE-G-Chi-Au/PtNP-ARV/MAb-BSA and GE-G-Chi-Au/PtNP-CH-ARV/MAb-BSA were used to detect ARV at a concentration of 10^6.82^ EID_50_ mL^−1^ to 10^0.82^ EID_50_ mL^−1^. The results are shown in Fig. 4. R_et_ increased with increasing concentrations of ARV due to the increased formation of the antigen–antibody complex, which is a nonconducting biomolecule. As observed, GE-G-Chi-Au/PtNP-Glu-ARV/MAb-BSA showed saturation beyond 10^3.82^ EID_50_ mL^−1^ ARV and a linear range of 0–10^3.82^ EID_50_ mL^−1^ ARV; the linear regression equation of the calibration curve was expressed as R_et_ (Ω) = 765 lgEID_50_ mL^−1^ + 1464 with a correlation coefficient of R^2^ = 0.96175, a low detection limit of 10^0.63^ EID_50_ mL^−1^ ARV (S/N = 3) and a quantification limit of 10^1.15^ EID_50_ mL^−1^ ARV (S/N = 10) (Fig. 4a,b). GE-G-Chi-Au/PtNP-EDC/NHS-ARV/MAb-BSA showed saturation beyond 10^3.82^ EID_50_ mL^−1^ ARV and a linear range of 0–10^3.82^ EID_50_ mL^−1^ ARV; the linear regression equation of the calibration curve was expressed as R_et_ (Ω) = 829 lgEID_50_ mL^−1^ + 2406 with a correlation coefficient of R^2^ = 0.98785, a low detection limit of 10^0.48^ EID_50_ mL^−1^ ARV (S/N = 3) and a quantification limit of 10^1.00^ EID_50_ mL^−1^ ARV (S/N = 10) (Fig. 4c,d). GE-G-Chi-Au/PtNP-ARV/MAb-BSA showed saturation beyond 10^4.82^ EID_50_ mL^−1^ ARV and a linear range of 0–10^4.82^ EID_50_ mL^−1^ ARV; the linear regression equation of the calibration curve was expressed as R_et_ (Ω) = 1123 lgEID_50_ mL^−1^ + 1938 with a correlation coefficient of R^2^ = 0.99347, a low detection limit of 10^0.37^ EID_50_ mL^−1^ ARV (S/N = 3) (Fig. 4e,f) and a quantification limit of 10^0.89^ EID_50_ mL^−1^ ARV (S/N = 10). GE-G-Chi-Au/PtNP-CH-ARV/MAb-BSA showed saturation beyond 10^5.82^ EID_50_ mL^−1^ ARV and a linear range of 0–10^5.82^ EID_50_ mL^−1^ ARV; the linear regression equation of the calibration curve was expressed as R_et_ (Ω) = 1084 lgEID_50_ mL^−1^ + 2335 with a correlation coefficient of R^2^ = 0.9673, a low detection limit of 10^0.46^ EID_50_ mL^−1^ ARV (S/N = 3) and a quantification limit of 10^0.98^ EID_50_ mL^−1^ ARV (S/N = 10) (Fig. 4g,h).

**Figure 5:**
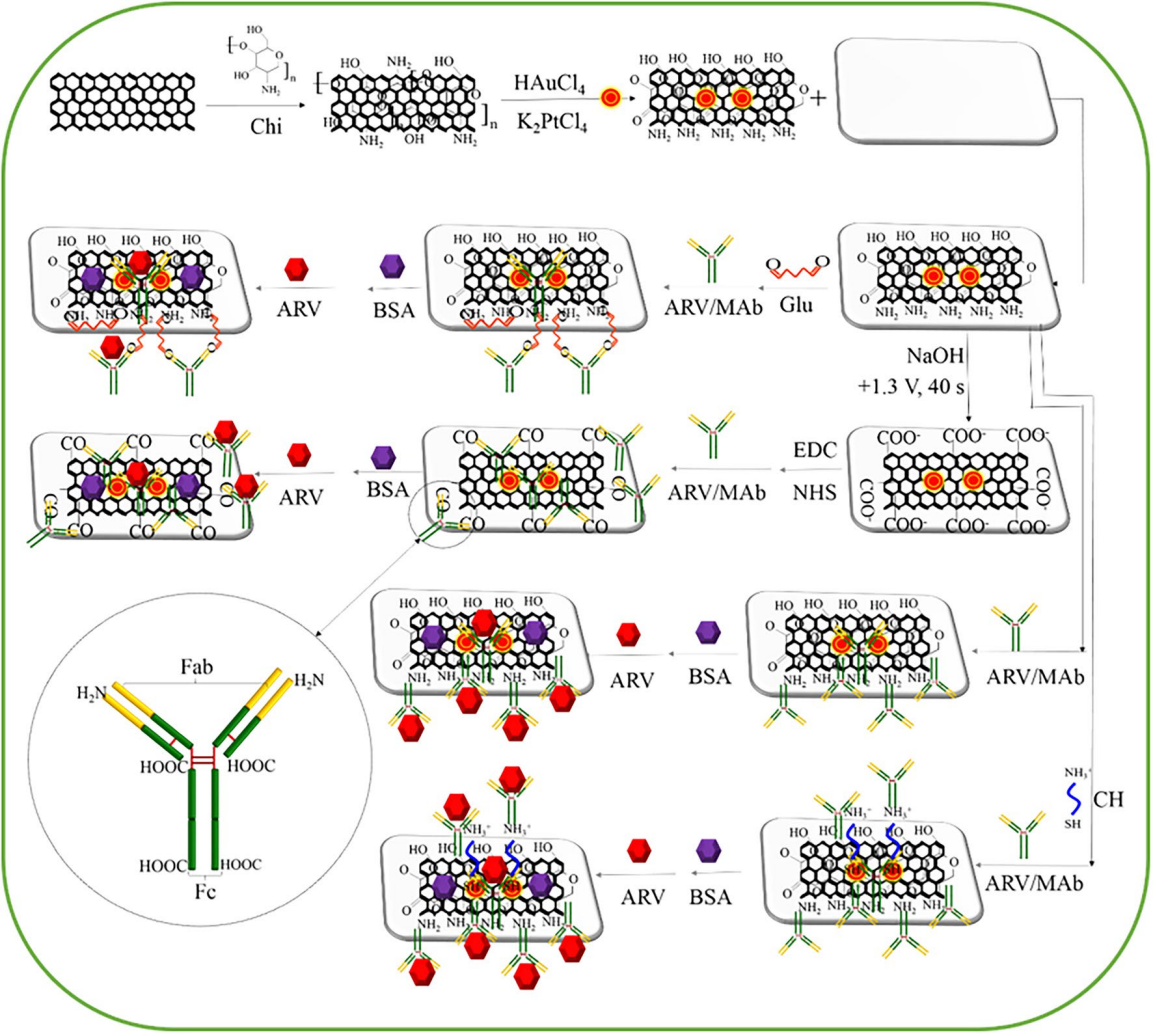
Schematic illustration of the electrochemical immunosensors.

These results show that the sensitivities of the four immunosensors were slightly different, while saturation beyond the extended dynamic range of GE-G-Chi-Au/PtNP-CH-ARV/MAb-BSA was 10 times that of GE-G-Chi-Au/PtNP-ARV/MAb-BSA and 100 times those of GE-G-Chi-Au/PtNP-Glu-ARV/MAb-BSA and GE-G-Chi-Au/PtNP-EDC/NHS-ARV/MAb-BSA. These results were attributed to the different methods of ARV/MAb immobilization. ARV/MAb is a Y-shaped protein that consists of two light and two heavy chains linked by disulfide bonds, and ARV/MAb is monomeric (IgG) and contains the F_ab_ region and F_c_ region (Fig. 5). The F_ab_ region, which participates in antigen binding, consists of an amino end, while the F_c_ region, which is the stem of the Y shape, has a carboxyl end group^29,30^. The region (F_ab_ or F_c_) of ARV/MAb attached to the electrode depended on immobilization strategy used. For GE-G-Chi-Au/PtNP-Glu-ARV/MAb-BSA, Glu was used as the coupling agent to connect amine groups on the surface of the electrode and amine groups (F_ab_ region) on ARV/MAb. The EDC/NHS-based immobilization strategy for GE-G-Chi-Au/PtNP-EDC/NHS-ARV/MAb-BSA activated carboxyl groups on the surface of the electrode and allowed NHS ester groups to act as intermediates leading to covalent attachment of the activated carboxyl groups with amino groups on the surface of the electrode present in the F_ab_ region on ARV/MAb. The antibodies in GE-G-Chi-Au/PtNP-ARV/MAb-BSA and GE-G-Chi-Au/PtNP-CH-ARV/MAb-BSA were immobilized onto the electrode via the tail end of the F_c_ region through covalent attachments between electrode surface amino groups and carboxyl groups on ARV/MAb, which resulted in accessibility for antigen binding because it was located in an orthogonal position. However, attachment of ARV/MAb onto the electrode through the F_ab_ region led to a conformation providing difficult antibody–antigen binding because of steric hindrance. Therefore, GE-G-Chi-Au/PtNP-ARV/MAb-BSA and GE-G-Chi-Au/PtNP-CH-ARV/MAb-BSA showed higher saturation levels for ARV than GE-G-Chi-Au/PtNP-Glu-ARV/MAb-BSA and GE-G-Chi-Au/PtNP-EDC/NHS-ARV/MAb-BSA, because GE-G-Chi-Au/PtNP-ARV/MAb-BSA and GE-G-Chi-Au/PtNP-CH-ARV/MAb-BSA provided more active sites for antigen binding when ARV was present in high concentration. Although antibodies of GE-G-Chi-Au/PtNP-ARV/MAb-BSA and GE-G-Chi-Au/PtNP-CH-ARV/MAb-BSA were immobilized onto the electrode through the F_c_ region, GE-G-Chi-Au/PtNP-CH-ARV/MAb-BSA showed a higher saturation level for ARV because it featured more amine groups with which to adsorb more ARV/MAb.

The sensitivity of an immunosensor depends on changes in charge transfer resistance per unit change in ARV concentration per unit area. The four different immunosensors were modified with GE-G-Chi-Au/PtNP. So their electron transfer abilities on the surfaces of the four different immunosensors were similar. In the detection process, antigen–antibody complex formation resulted in similar changes in charge transfer resistance per unit change in ARV concentration per unit area, which provided similar sensitivities for the four different immunosensors. While the different strategies for antibody immobilization led to slight sensitivity differences among the four different immunosensors, GE-G-Chi-Au/PtNP-ARV/MAb-BSA showed the highest sensitivity among the four different immunosensors because ARV/MAb was immobilized on the surface of electrode by direct incubation (we did not use any non-conducting organics as linkers); ARV/MAb in the other three immunosensors was immobilized on the electrode surface with Glu, EDC/NHS and CH as linker, respectively. Glu, EDC/NHS and CH with poor electrical conductivities were attached to the electrodes, and the electron transfer abilities decreased, so the sensitivities of the immunosensors were decreased.

### Selectivity, reproducibility and stability of the four immunosensors

**Figure 6:**
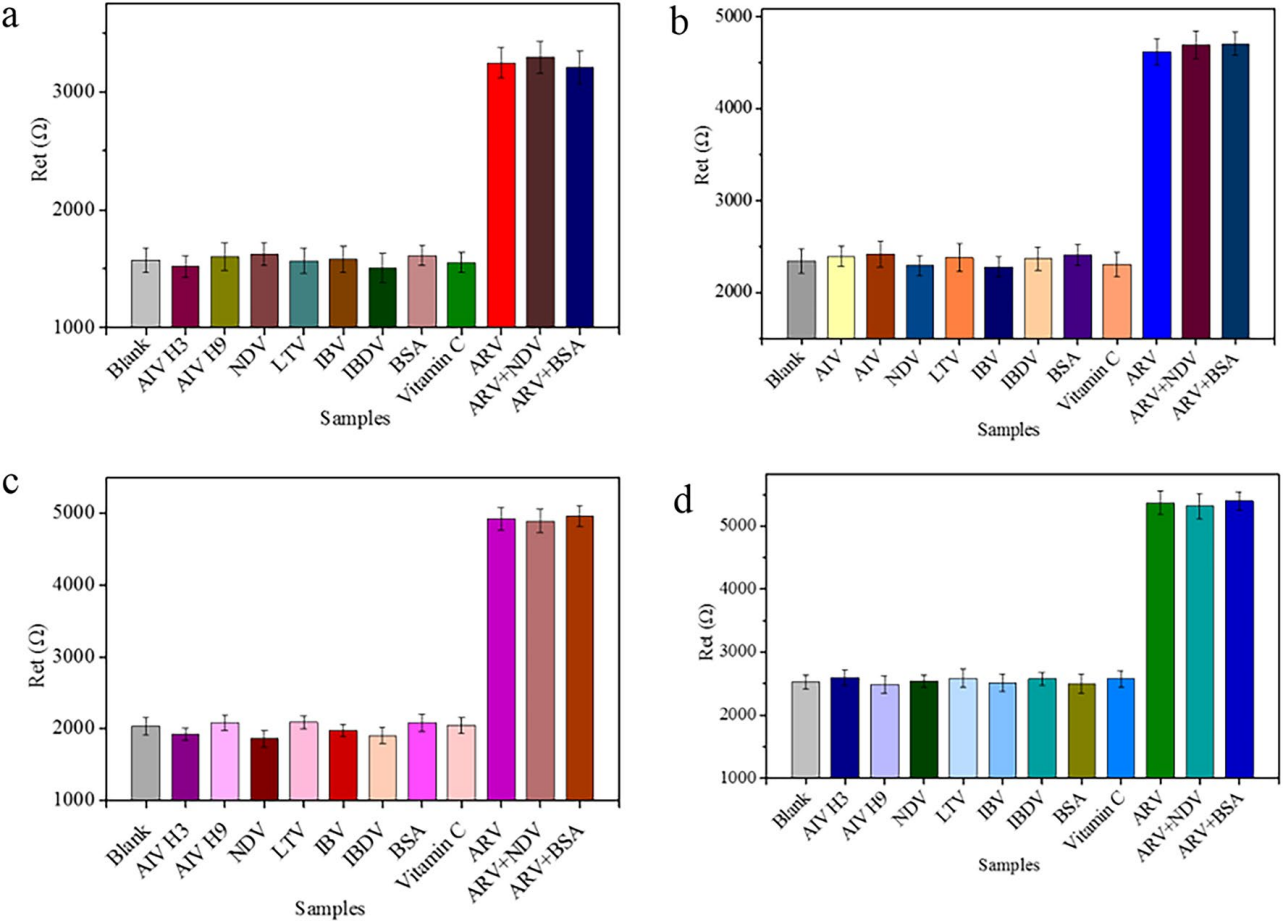
Specificity of the immunosensors toward the target (ARV) and other interfering substances. (**a**) GE-G-Chi-Au/PtNP-Glu-ARV/MAb-BSA, (**b**) GE-G-Chi-Au/PtNP-EDC/NHS-ARV/MAb-BSA, (**c**) GE-G-Chi-Au/PtNP-ARV/MAb-BSA, (**d**) GE-G-Chi-Au/PtNP-CH-ARV/MAb-BSA. Error bar = RSD (n = 5).

The selectivity of GE-G-Chi-Au/PtNP-Glu-ARV/MAb-BSA, GE-G-Chi-Au/PtNP-EDC/NHS-ARV/MAb-BSA, GE-G-Chi-Au/PtNP-ARV/MAb-BSA and GE-G-Chi-Au/PtNP-CH-ARV/MAb-BSA played a crucial role in detecting target samples without separation. To evaluate the selectivities of the four different immunosensors, avian influenza virus H3 subtype (AIV H3, 10^4.71^ EID_50_ mL^−1^), avian influenza virus H9 subtype (AIV H9, 10^3.74^ EID_50_ mL^−1^), Newcastle disease virus (NDV, 10^4.53^ EID_50_ mL^−1^), laryngotracheitis virus (LTV, 10^3.86^ EID_50_ mL^−1^), infectious bronchitis virus (IBV, 10^4.36^ EID_50_ mL^−1^), infectious bursal disease virus (IBDV, 10^4.67^ EID_50_ mL^−1^), BSA (1.0 μg/mL) and vitamin C (1.0 μg/mL) were used as interfering substances. As shown in Fig. 6, the R_et_s of the samples with the interfering substances were almost the same as that of the blank control, and the mixtures of ARV (10^2.82^ EID_50_ mL^−1^) with other possible interfering substances showed similar R_et_ values to those of ARV (10^2.82^ EID_50_ mL^−1^), indicating that GE-G-Chi-Au/PtNP-Glu-ARV/MAb-BSA, GE-G-Chi-Au/PtNP-EDC/NHS-ARV/MAb-BSA, GE-G-Chi-Au/PtNP-ARV/MAb-BSA and GE-G-Chi-Au/PtNP-CH-ARV/MAb-BSA had high selectivity for ARV detection.

**Figure 7:**
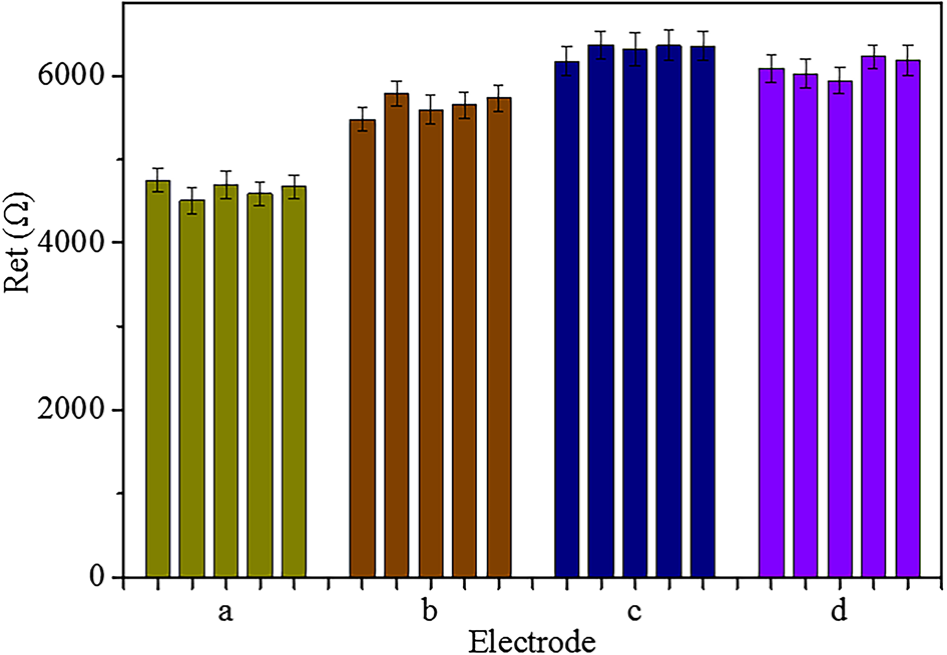
Reproducibility of the four immunosensors in the presence of 10^3.82^ EID_50_ mL^−1^ ARV (a) GE-G-Chi-Au/PtNP-Glu-ARV/MAb-BSA, (b) GE-G-Chi-Au/PtNP-EDC/NHS-ARV/MAb-BSA, (c) GE-G-Chi-Au/PtNP-ARV/MAb-BSA, (d) GE-G-Chi-Au/PtNP-CH-ARV/MAb-BSA. Error bar = RSD (n = 5).

The reproducibility of GE-G-Chi-Au/PtNP-Glu-ARV/MAb-BSA, GE-G-Chi-Au/PtNP-EDC/NHS-ARV/MAb-BSA, GE-G-Chi-Au/PtNP-ARV/MAb-BSA and GE-G-Chi-Au/PtNP-CH-ARV/MAb-BSA was investigated by EIS at the same concentration of 10^3.82^ EID_50_ mL^−1^ ARV. The results are shown in Fig. 7. The RSDs of GE-G-Chi-Au/PtNP-Glu-ARV/MAb-BSA, GE-G-Chi-Au/PtNP-EDC/NHS-ARV/MAb-BSA, GE-G-Chi-Au/PtNP-ARV/MAb-BSA and GE-G-Chi-Au/PtNP-CH-ARV/MAb-BSA were 2.04%, 2.12%, 1.30% and 1.92%, respectively, indicating that the four immunosensors have good reproducibility.

**Figure 8:**
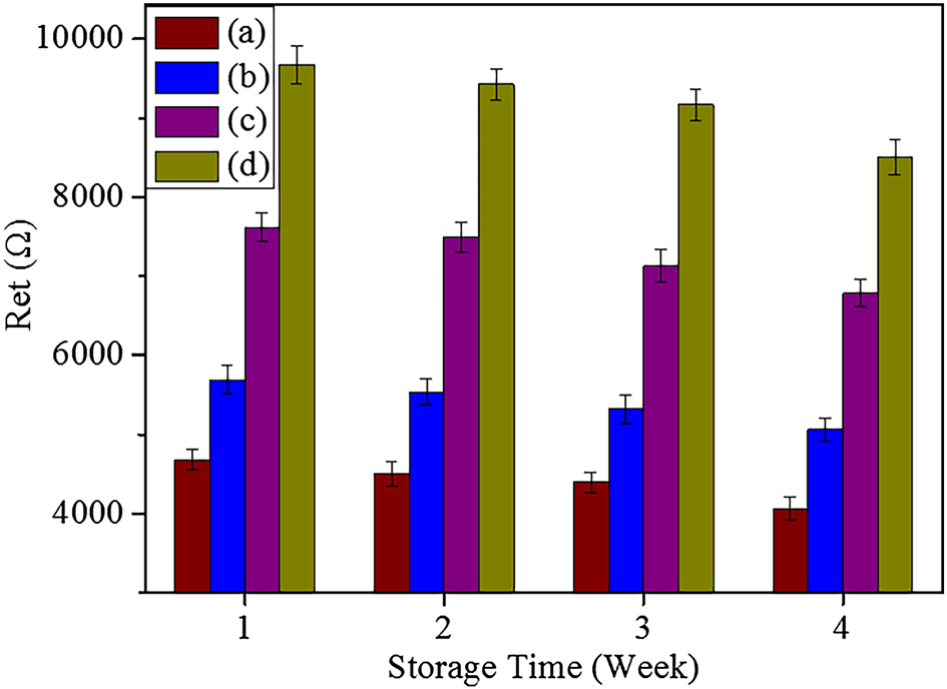
Long-term stability of the proposed immunosensors: (a) GE-G-Chi-Au/PtNP-Glu-ARV/MAb-BSA, (b) GE-G-Chi-Au/PtNP-EDC/NHS-ARV/MAb-BSA, (c) GE-G-Chi-Au/PtNP-ARV/MAb-BSA, (d) GE-G-Chi-Au/PtNP-CH-ARV/MAb-BSA.

The long-term stability of the fabricated electrochemical immunosensors was further investigated by storing GE-G-Chi-Au/PtNP-Glu-ARV/MAb-BSA, GE-G-Chi-Au/PtNP-EDC/NHS-ARV/MAb-BSA, GE-G-Chi-Au/PtNP-ARV/MAb-BSA and GE-G-Chi-Au/PtNP-CH-ARV/MAb-BSA at 4 °C when not in use and then successively incubating them with ARV (10^5.82^ EID_50_ mL^−1^). As seen in Fig. 8, the R_et_s remained at 91.72%, 90.39%, 93.07%, and 92.31% of the initial values when GE-G-Chi-Au/PtNP-Glu-ARV/MAb-BSA, GE-G-Chi-Au/PtNP-EDC/NHS-ARV/MAb-BSA, GE-G-Chi-Au/PtNP-ARV/MAb-BSA and GE-G-Chi-Au/PtNP-CH-ARV/MAb-BSA were stored at 4 °C for 4 weeks. The results indicated that the long-term stability of the four electrochemical immunosensors for ARV detection was acceptable.

## Conclusions

In this work, four different antibody immobilization strategies (Glu as a coupling agent to connect amine groups on the surface of the electrode and amine groups present in ARV/Mab; EDC/NHS chemistry to activate carboxyl groups on the surface of the electrode for covalent attachment with amine groups present at the F_ab_ region on ARV/Mab; immobilization of ARV/MAb directly onto the amine group ending the electrode; and immobilization of ARV/MAb directly onto the amine group ending the electrode after it was further modified with CH) were used to construct four different immunosensors (GE-G-Chi-Au/PtNP-Glu-ARV/MAb-BSA, GE-G-Chi-Au/PtNP-EDC/NHS-ARV/MAb-BSA, GE-G-Chi-Au/PtNP-ARV/MAb-BSA and GE-G-Chi-Au/PtNP-CH-ARV/MAb-BSA) to study the effect of immobilization strategy on the immunosensor performance. The EIS results showed that the extended dynamic range of GE-G-Chi-Au/PtNP-CH-ARV/MAb-BSA was 10 times that of GE-G-Chi-Au/PtNP-ARV/MAb-BSA and 100 times that of GE-G-Chi-Au/PtNP-Glu-ARV/MAb-BSA and GE-G-Chi-Au/PtNP-EDC/NHS-ARV/MAb-BSA, indicating that the immunosensors that immobilized the antibody via the F_c_ region obtained an extended dynamic response. In addition, the sensitivities, selectivities, reproducibilities and stabilities of the immunosensors were almost completely unaffected by the antibody immobilization strategies used in this work.

## Methods

Bovine serum albumin (BSA), chloroauric acid tetrahydrate (HAuCl_4_·4H_2_O) and chloroplatinic acid hexahydrate (H_2_PtCl_6_·6H_2_O) were procured from Sigma–Aldrich Chemical Co. (St. Louis, MO, USA.). Graphite powder (< 45 mm), potassium ferrocyanide (K_4_[Fe(CN)_6_]), potassium ferricyanide (K_3_[Fe(CN)_6_]), Glu, EDC, NHS, CH, H_2_SO_4_ and KMnO_4_ were obtained from Guoyao Group Chemical Reagents Co., Ltd. (Shanghai, China).

### Viruses and antibodies

ARV (S1133, China Institute of Veterinary Drug Control), AIV H3 (A/Duck/Guangxi/M20/2009, Guangxi Veterinary Research Institute), AIV H9 (A/Chicken/Guangxi/DX/2008, Guangxi Veterinary Research Institute), NDV (F48E9, China Institute of Veterinary Drug Control), LTV (ILT/13, China Institute of Veterinary Drug Control), IBV (Mass41, China Institute of Veterinary Drug Control) and IBDV (China Institute of Veterinary Drug Control) were collected and stored in a − 80 °C freezer in our laboratory prior to use. ARV/MAbs were prepared by our group^31^.

### Synthesis of G-Chi-Au/PtNP-ARV/MAbs

First, G was obtained by the Hummers method with slight modifications^32^. In short, 2.5 g NaNO_3_ and 1.0 g graphite powder were added to 100 mL concentrated H_2_SO_4_ under continuous stirring and reacted at room temperature for 2 h. Then, the obtained mixture was cooled in an ice bath, 5 g KMnO_4_ was slowly added under continuous stirring, and the mixture was kept for 24 h at 35 °C. Then, 100 mL ddH_2_O was added to the mixture under continuous stirring, the mixture was kept for 3 h at 80 °C, and 300 mL ddH_2_O was added. Subsequently, 6 mL 30% H_2_O_2_ was added, the mixture solution turned bright yellow, and many bubbles appeared. After continuous stirring for 3 h, the obtained solution was precipitated for 24 h at room temperature, the supernatant was decanted, 500 mL 0.5 mol/L HCl was added to the slurry, and the mixture was washed by centrifugation. Next, the obtained slurry was washed with ddH_2_O until the pH of the supernatant was approximately 7.0. Then, 100 mL ddH_2_O was added, and G oxide was obtained after ultrasonication for 2 h. The G oxide was heated to 95 °C in a water bath, 10 mL 1.0% NaBH_4_ was added under continuous stirring, and the mixture was kept for 3 h, washed with ddH_2_O three times, and dried in a vacuum drying oven at 90 °C for 8 h to obtain G.

Second, G-Chi was prepared according to our previous report^33^. Briefly, 0.05 mg Chi powder was added to 100 mL 1.0% (v/v) acetic acid solution under continuous stirring at room temperature and maintained for 0.5 h. Then, 100 mg of G was added, the mixture was continuously stirred for 24 h, and G-Chi was collected by centrifugation and washed with dd-H_2_O.

Third, 1 mL 10 mmol/L HAuCl_4_, 1 mL 10 mmol/L K_2_PtCl_4_ and 20 mL 1 mg/mL G-Chi solution were mixed together, and the mixture was stirred at room temperature for 3 h. Then, the mixture was heated to 80 °C under continuous stirring for 1 h to obtain the G-Chi-Au/PtNP nanocomposite.

### Fabrication of the electrochemical immunosensor

The step-by-step fabrication of the electrochemical immunosensor is illustrated in Fig. 5. A gold electrode (GE) was polished with 1.0 μm, 0.3 μm, and 0.05 μm alumina polishing powders, rinsed with ddH_2_O, and cleaned by sonication in ddH_2_O, ethanol, and ddH_2_O for 5 min each. Subsequently, the GE was dried by flushing with nitrogen gas.

Next, 8 μL of prepared G-Chi-Au/PtNP was dropped onto the surface of the clean GE and dried at room temperature, and G-Chi-Au/PtNP-GE was obtained. ARV/MAbs was immobilized onto G-Chi-Au/PtNP-GE by four different strategies: (1) G-Chi-Au/PtNP-GE was incubated with 10 μL of 5% Glu for 3 h and washed with PBS three times, and then 8 μL of 100 μg/mL ARV/MAbs was deposited onto G-Chi-Au/PtNP-Glu-GE and incubated at 4 °C for 8 h; (2) G-Chi-Au/PtNP-GE was anodized in 0.5 mol/L NaOH solution with a potential of + 1.3 V for 40 s to increase the number of –COOH groups on its surface. The anodized G-Chi-Au/PtNP-GE was incubated with 10 μL of solution that contained 50 mmol/L EDC and 30 mmol/L NHS in MES (pH 4.7) at room temperature for 1 h. –COOH groups were converted to amine-reactive NHS esters in this step to prepare for ARV/mAb immobilization. Then, after washing with PBS (pH 7.4) three times to remove the unreacted EDC/NHS, 8 μL 100 μg/mL ARV/MAbs was deposited onto the NHS-activated surface of G-Chi-Au/PtNP-EDC/NHS-GE, which was then incubated at 4 °C for 8 h; (3) 8 μL 100 μg/mL ARV/MAbs was deposited onto G-Chi-Au/PtNP-GE and incubated at 4 °C for 8 h without any further modification; and (4) G-Chi-Au/PtNP-GE was incubated with 10 μL 2 mg/mL CH at room temperature in the dark for 4 h and washed with PBS three times, after which 8 μL 100 μg/mL ARV/MAbs was deposited onto G-Chi-Au/PtNP-CH-GE, and the material was incubated at 4 °C for 8 h. All the electrodes with immobilized ARV/MAbs prepared via above four strategies were washed with PBS (pH 7.4) to remove physically adsorbed or excess ARV/MAbs, incubated with 1% BSA in 0.01 mol/L PBS (pH 7.4) at room temperature for 1 h to block the free active sites on the electrodes and washed three time with PBS (pH 7.4). The obtained immunosensors were denoted GE-G-Chi-Au/PtNP-Glu-ARV/MAb-BSA, GE-G-Chi-Au/PtNP-EDC/NHS-ARV/MAb-BSA, GE-G-Chi-Au/PtNP-ARV/MAb-BSA and GE-G-Chi-Au/PtNP-CH-ARV/MAb-BSA, respectively, and stored at 4 °C until use.

### Electrochemical analysis

The immunosensors were incubated with 8 μL of different concentrations of ARV at 37 °C for 30 min and washed with PBS (pH 7.4). Then, the sensors were subjected to EIS measurements in buffer containing 5.0 mM K_3_[Fe(CN)]_6_ and K_4_[Fe(CN)]_6_ in PBS (pH 7.4).

## Data Availability

All data generated or analyzed during this study are included in this article.
